# A case series of the twiddler syndrome

**DOI:** 10.1093/ehjcr/ytae004

**Published:** 2024-01-05

**Authors:** Roberta Montisci, Cinzia Soro, Roberta Demelas, Elena Agus, Alessio Follesa, Gesualdo Siragusa, Vincenzo Nissardi

**Affiliations:** Cardiologia-UTIC, Policlinico ‘D. Casula’, Azienda Ospedaliero-Universitaria—Cagliari, Department of Clinical Sciences and Public Health, University of Cagliari, SS. 554, Km 4,5, Monserrato, Cagliari 09042, Italy; Cardiologia-UTIC, Policlinico ‘D. Casula’, Azienda Ospedaliero-Universitaria—Cagliari, Department of Clinical Sciences and Public Health, University of Cagliari, SS. 554, Km 4,5, Monserrato, Cagliari 09042, Italy; Cardiologia-UTIC, Struttura Semplice di Cardiostimolazione, Policlinico ‘D. Casula’, Azienda Ospedaliero-Universitaria—Cagliari, SS. 554, Km 4,5, Monserrato, Cagliari 09042, Italy; Cardiologia-UTIC, Policlinico ‘D. Casula’, Azienda Ospedaliero-Universitaria—Cagliari, Department of Clinical Sciences and Public Health, University of Cagliari, SS. 554, Km 4,5, Monserrato, Cagliari 09042, Italy; Cardiologia-UTIC, Policlinico ‘D. Casula’, Azienda Ospedaliero-Universitaria—Cagliari, Department of Clinical Sciences and Public Health, University of Cagliari, SS. 554, Km 4,5, Monserrato, Cagliari 09042, Italy; Cardiologia-UTIC, Policlinico ‘D. Casula’, Azienda Ospedaliero-Universitaria—Cagliari, Department of Clinical Sciences and Public Health, University of Cagliari, SS. 554, Km 4,5, Monserrato, Cagliari 09042, Italy; Cardiologia-UTIC, Policlinico ‘D. Casula’, Azienda Ospedaliero-Universitaria—Cagliari, Department of Clinical Sciences and Public Health, University of Cagliari, SS. 554, Km 4,5, Monserrato, Cagliari 09042, Italy; Cardiologia-UTIC, Struttura Semplice di Cardiostimolazione, Policlinico ‘D. Casula’, Azienda Ospedaliero-Universitaria—Cagliari, SS. 554, Km 4,5, Monserrato, Cagliari 09042, Italy; Cardiologia-UTIC, Policlinico ‘D. Casula’, Azienda Ospedaliero-Universitaria—Cagliari, Department of Clinical Sciences and Public Health, University of Cagliari, SS. 554, Km 4,5, Monserrato, Cagliari 09042, Italy; Cardiologia-UTIC, Struttura Semplice di Cardiostimolazione, Policlinico ‘D. Casula’, Azienda Ospedaliero-Universitaria—Cagliari, SS. 554, Km 4,5, Monserrato, Cagliari 09042, Italy

**Keywords:** Twiddler syndrome, Leadless pacemaker, Submuscular pacemaker implant, Case series

## Abstract

**Background:**

Twiddler syndrome (TS) is a complication of cardiac implantable electronic device (CIED) implantation, caused by the deliberate or unconscious manipulation of the device by the patient himself, which results in dislocation of the leads by retraction towards the subcutaneous pocket.

**Case summary:**

This report describes two clinical cases that occurred in our centre, for which two different solutions were successfully implemented. In the first case, a complete removal of the stimulation system was performed, and a leadless pacemaker (PM; Medtronic Micra VR) was implanted. In the second case, the patient underwent a revision procedure. The PM was disconnected, and the electrodes were debrided, a submuscular pocket for the PM was created, and at the end of the procedure, the PM was anchored to the pectoralis major.

**Discussion:**

Twiddler syndrome is a not so rare and serious complication of CIED implantation, leading to device malfunctioning and higher risk of infection of the pocket due to multiple re-interventions. In these two cases, different surgical solutions were performed, both resulting to be effective to solve the effects of TS.

**Learning points**

- Twiddler syndrome is a not so rare complication (0.7–7%) following the implant of any cardiac implantable electronic device due to the deliberate or unconscious manipulation of the device by the patient himself, which causes malfunction of implanted system.
- It is important to recognize it early in order to prevent dislocation of the device and to act early in a definitive manner.
- We have adopted two different surgical solutions to avoid manipulation of the devices by patients.
- At the time of device implantation, a psychological evaluation can be useful to identify patients predisposed to twiddler syndrome in order to be able to prevent it.

## Introduction

Twiddler syndrome (TS), first described by Bayliss *et al.*^1^ in 1968, is a complication (incidence 0.07–7%)^2^ following the implant of any cardiac implantable electronic device (CIED) due to the deliberate or unconscious manipulation of the device by the patient himself, which causes the dislocation of the leads by retraction towards the subcutaneous pocket.^3^ In most cases, it occurs within the first year of implantation of the device.^2^

Lead dislocation can result in interruption of pacing and sensing functions. The progression of the retraction can cause the stimulation of the ipsilateral phrenic nerve, the brachial plexus, or the chest wall muscles.^3,4^

Risk factors include female gender, obesity, older age due to increased subcutaneous tissue laxity, cognitive impairment, and the discrepancy between the size of the subcutaneous pocket compared with the size of the device used.^3,5^

Two TS clinical cases are described, which were solved using two different procedural solutions (Supplementary material online).

## Summary figure

**Figure:**
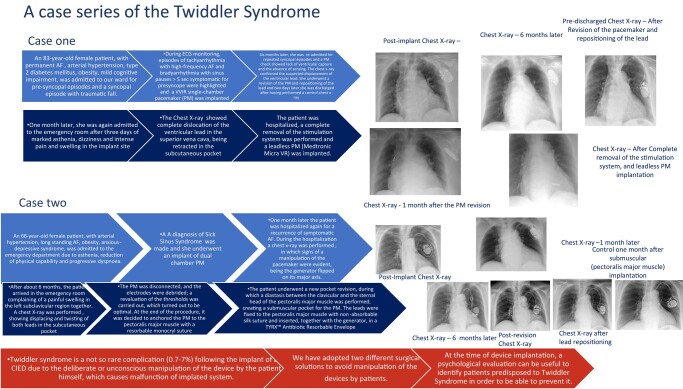


## Case report 1

An 83-year-old female patient, with permanent atrial fibrillation (AF) on direct oral anticoagulant (DOAC) therapy, arterial hypertension, type 2 diabetes mellitus, obesity, and mild cognitive impairment, was admitted to our ward for the occurrence of many pre-syncopal episodes not related to physical exertion and not preceded by prodrome and a syncopal episode with traumatic fall. Her blood pressure was 134/80 mmHg, and her pulse rate was 130/min, irregular. No pathological findings on cardiac and chest examination and no oedema of the lower legs. During hospitalization, the continuous electrocardiographic monitoring showed the alternance of rapid AF with average heart rate (HR) above 130 b.p.m., perceived by the patient with palpitation, dyspnoea and discomfort, and repeated episodes of AF bradyarrhythmia with pauses up to 5 s, during both day and night, causing dizziness despite the supine position.

On the 15th day, a VVIR single-chamber pacemaker (PM) was implanted.

**Figure 1:**
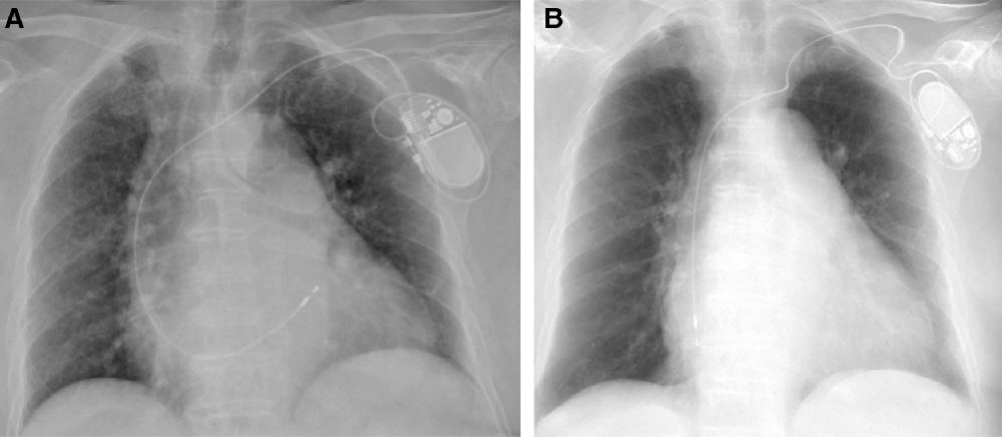
Case report 1. (*A*) Post-implant chest X-ray. (*B*) Chest X-ray—6 months later.

The patient was discharged 2 days later in a generally good condition, after having performed a post-implantation chest X-ray (*Figure 1A*).

A month later, she underwent a device check that confirmed good pacing and sensing thresholds.

**Figure 2:**
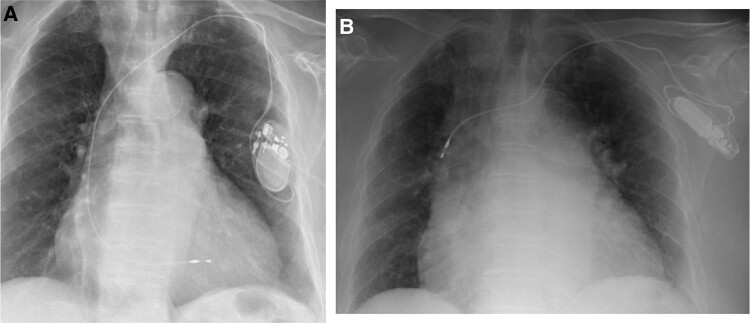
Case report 1. (*A*) Pre-discharged chest X-ray—after revision of the pacemaker and repositioning of the lead. (*B*) Chest X-ray—1 month after the pacemaker revision.

Six months later, she was admitted to the emergency department, complaining of having repeating pre-syncopal episodes, during the last few days, associated with a decline in her physical condition. A PM interrogation was performed, which showed lack of ventricular capture and the absence of sensing, together with a chest X-ray (*Figure 1B*), that confirmed the suspected displacement of the ventricular lead. She underwent a revision of the PM and repositioning of the lead, and 2 days later, she was discharged after having performed a control chest X-ray (*Figure 2A*).

**Figure 3:**
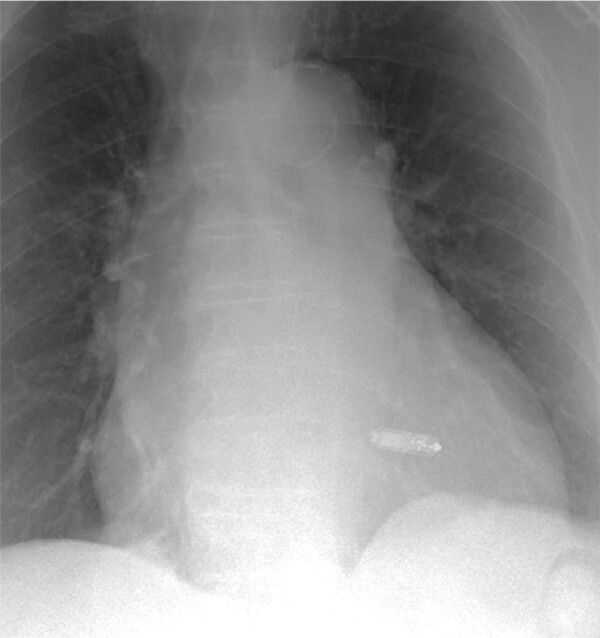
Case report 1. Chest X-ray—after complete removal of the stimulation system and leadless pacemaker (Medtronic Micra VR) implantation.

One month later, she was again admitted to the emergency room after 3 days of marked asthenia, dizziness, and intense pain and swelling in the implant site. During the visit, the patient continued to rub her PM pocket in a completely unconscious way; in fact, when she was told not to touch the PM, the patient denied it completely. The patient suffered from mild cognitive impairment and was probably unable to understand the consequences, and a psychiatric evaluation was offered to the patient who declined. A chest X-ray was performed, which showed complete dislocation of the ventricular lead in the superior vena cava, being retracted in the subcutaneous pocket (*Figure 2B*). The patient was hospitalized, a complete removal of the stimulation system was performed, and a leadless PM (Medtronic Micra VR) was implanted. On the 13th day of hospitalization, the patient was discharged after performing a new chest X-ray (*Figure 3*).

During the following check-ups, about 6 months after the leadless PM implant, optimal pacing and sensing thresholds were confirmed.

## Case report 2

A 68-year-old female patient, with history of arterial hypertension since menopause, long-standing AF treated with DOAC, obesity, and anxious–depressive syndrome, was admitted to the emergency department due to asthenia, reduction of physical capability, and progressive dyspnoea. The patient presented with a blood pressure of 171/80 mmHg and a regular heart rate of 45 b.p.m. on admission. On physical examination, there are no heart murmurs, there are fine rales at the lung bases, and no oedema of the lower limbs. The electrocardiogram (ECG) showed moderate sinus bradycardia with HR 46/min. During hospitalization, alternance of episodes of rapid AF (110 b.p.m.) and moderate to severe sinus bradycardia were found, with several episodes of sinus arrest resulting in pathological pauses (up to 7.6 s) symptomatic for pre-syncope. After diagnosis of sick sinus syndrome (bradycardia–tachycardia syndrome), considering the need of starting rhythm control therapy for the recurrence of AF, on the fourth day, the patient underwent a dual-chamber PM implant, using passive-fixation leads. The post-implantation ECG documented the correct functioning of the PM.

**Figure 4:**
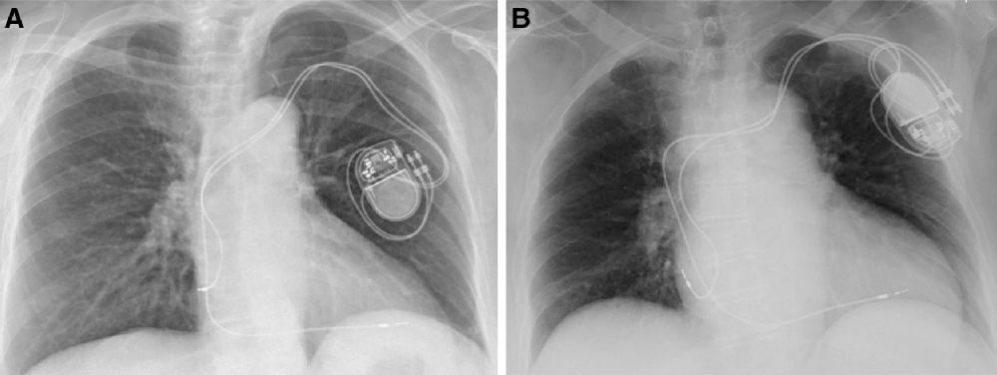
Case report 2. (*A*) Post-implant chest X-ray. (*B*) Chest X-ray—1 month later.

On the sixth day, the patient was discharged in good clinical condition after performing a post-procedural chest X-ray (*Figure 4A*).

One month later, the patient was hospitalized again for a recurrence of rapid AF symptomatic for palpitations. During the hospitalization, a chest X-ray was performed (*Figure 4B*), in which signs of a manipulation of the PM were evident, being the generator flipped on its major axis. We advised the patient not to manipulate the PM pocket, and we offered to the patient a psychiatric counselling, but she refused as she was already being followed up for her anxious–depressive syndrome.

**Figure 5:**
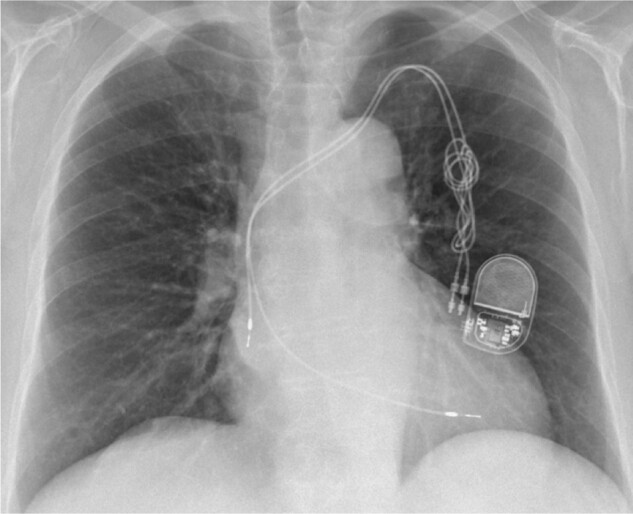
Case report 2. Chest X-ray—7 months later.

**Figure 6:**
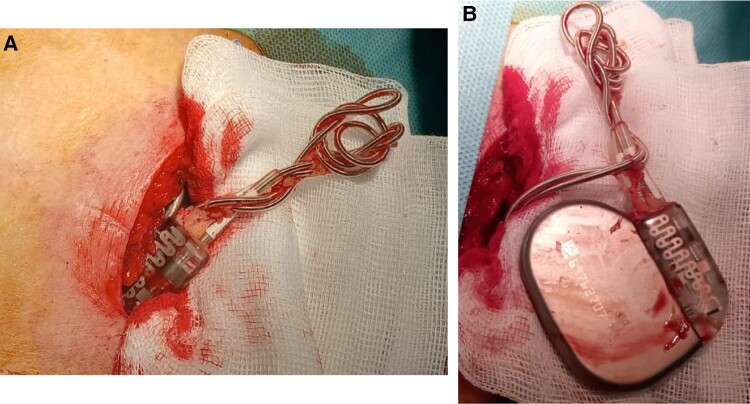
Case report 2. (*A*, *B*) Phases of the lead repositioning surgery.

After about 6 months, the patient arrived in the emergency room complaining of a painful swelling in the left subclavicular region. A chest X-ray was performed (*Figure 5*), showing displacing and twisting of both leads in the subcutaneous pocket. In consideration of both lead retractions, the patient underwent a revision procedure. When the generator was extracted, a kinking of the leads was found (*Figure 6A* and *B*). The PM was disconnected, and the electrodes were debrided; a revaluation of the thresholds was carried out, which turned out to be optimal. At the end of the procedure, it was decided to anchor the PM to the pectoralis major muscle with a resorbable Monocryl suture.

**Figure 7:**
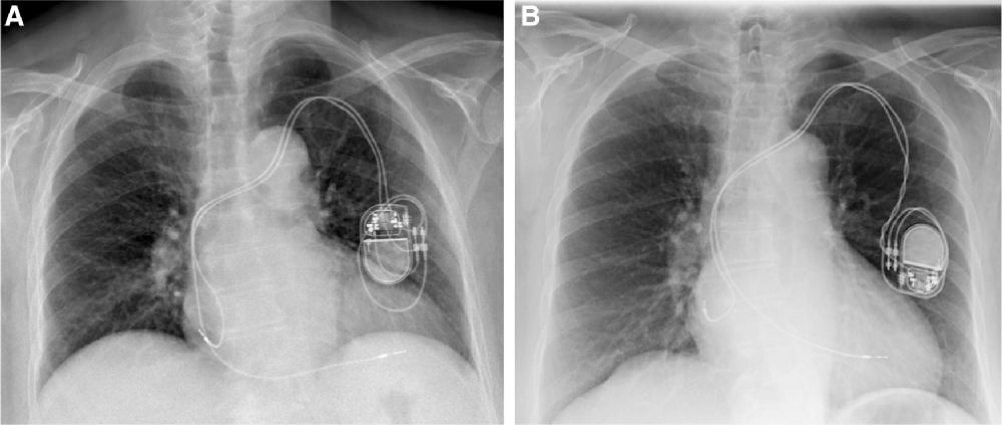
Case report 2. (*A*) Post-revision chest X-ray. (*B*) Chest X-ray after lead repositioning.

The patient was discharged 2 days later after post-procedural chest X-ray showing good lead positioning (*Figure 7A*).

One month after discharge, the patient underwent a new chest X-ray with evidence of initial rotation of the generator and a stretch of the ventricular lead, in the absence of a major dislocation and/or malfunction of the device (*Figure 7B*).

**Figure 8:**
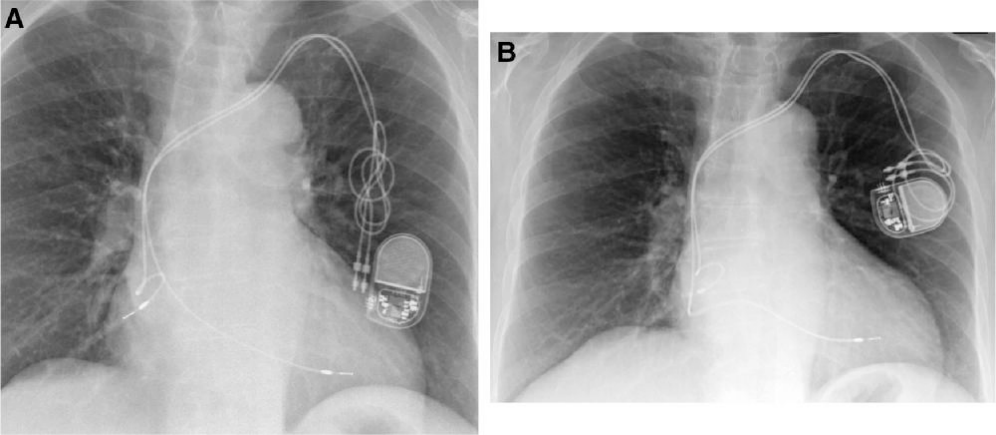
Case report 2. (*A*) Chest X-ray—3 months after the revision of pacing system. (*B*) Control chest X-ray—1 month after submuscular (pectoralis major muscle) implantation.

At the scheduled follow-up control, the patient complained again of painful swelling in the left subclavicular region. A check of the device was performed, which showed optimal thresholds, but because palpation of the area revealed a mass, a new chest X-ray was performed (*Figure 8A*). Stretching of the ventricular lead and twisting of both leads in the subclavicular area forming a knot were shown, with caudal sliding of the generator.

The patient underwent a new pocket revision 3 months after the first revision procedure, during which a diastasis between the clavicular and the sternal head of the pectoralis major muscle was performed, creating a submuscular pocket for the PM. The leads were fixed to the pectoralis major muscle with non-absorbable silk suture and inserted, together with the generator, in a TYRX™ Antibiotic Resorbable Envelope; the submuscular pocket was then closed with an absorbable Monocryl suture.

Two weeks later, the follow-up chest X-ray showed good positioning of the leads.

The patient underwent a new radiological control, 1 month later, which was unchanged compared with the previous one (*Figure 8B*).

After 2 months, the PM and the leads appeared to be stable and well positioned, excellent pacing and sensing thresholds persist, and the impedance of the leads did not show any changes. With the submuscular implant, the patient was no longer able ‘to twiddle’ the device.

## Discussion

Both patients consistently denied ‘twiddling’ their devices. This attitude went so far as to deny the evidence of self-manipulation, which took place in front of the doctor during the follow-up visits.

In two recently published case reports, a correlation has been made between obsessive–compulsive disorder (OCD) and TS,^6,7^ but in both cases, patients underwent the implant of neurological/brain stimulation devices in order to treat neurological and psychiatric disorders.

Another report^8^ describes the case of a 47-year-old patient undergoing implantable cardioverter defibrilator (ICD) implantation in secondary prevention, who developed a stress-induced OCD related to heart disease and fear of a possible shock from the ICD, leading to a compulsive manipulation of the device. The patient was treated with antidepressant oral medications and behavioural psychotherapy, with progressive resolution of the clinical condition.

According to the authors, sometimes patients tend to manipulate the implanted device in the early post-implantation period, as an automatic reflex to relieve pain due to the scarring process and/or to verify the integrity of the device, as an adaptive behaviour to the foreign object. Such manipulations seem to cease rapidly in patients with no previous psychiatric history and a good level of awareness of their disease; on the contrary, patients with psychiatric disorders and/or having little awareness of their disease usually keep on manipulating the device.^8^

In the same report,^8^ the authors suggest that introducing the clinical practice of a biopsychosocial approach, which includes a psychiatric evaluation of the patient undergoing the implantation of CIED, could be useful to identify patients at risk of TS, by guiding clinicians in the choice of the type of device to be implanted in the implementation of surgical manoeuvres to reduce the possibility of manipulation, or possibly in starting a psychiatric therapy.

In the two clinical cases presented, the patients both had numerous predisposing factors for TS: they were both obese and of an elderly age, both conditions being related to laxity of the subcutaneous tissues; in the case of patient 1, there was also a mild cognitive impairment. However, they did not show signs of OCD during hospitalizations or follow-up visits, apart from persistent self-manipulation of the devices. In the case of the patient 2, the anxious–depressive syndrome was well controlled by the drug therapy and paradoxically manifested itself with the fear of PM malfunction, which was induced by the manipulations itself.

Could a pre-intervention psychiatric evaluation reveal an OCD that could make us foresee the possible development of a TS? Could a psychotherapy approach following the implant be useful to reduce the manipulatory attitude? This approach was proposed to both patients and their families but rejected in both cases.

However, having found two satisfactory surgical solutions for TS bypassed the need for a psychiatric approach, which both patients and family members rejected.

We adopted two different solutions in the two settings, given the two different forms of sick sinus syndrome. In the case of Patient 1, the presence of permanent AF and advanced age allowed for the use of the single-chamber VVIR PM leadless. In the case of Patient 2, the younger age and the alternance of sinus rhythm and AF episodes led us to choose a traditional DDDR PM, due to the need to maintain atrial stimulation. The submuscular implant is simple and free from major complications in expert hands. During the procedure, the TYRX™ resorbable antibiotic envelope was used to reduce the risk of infection that is high in repeated interventions. We inserted in the envelope both the generator and the leads in order to minimize the possibility of any further manipulations, paying particular attention to anchor the tract of the lead before its insertion in the left subclavian vein to the pectoralis major muscle with non-absorbable silk stitches and the appropriate sleeves.

In both cases, the definitive solution was done after several revisions for different reasons. In Case 1, because the onset of the problem was slow and progressive, furthermore, the high cost of the leadless PM required longer bureaucratic processes. In Case 2, the lively denial of the manipulative activity by the patient and her family played an important role, so the diagnosis of TS was made more late; only when the manipulation activity took place in front of a doctor during follow-up was it decided to proceed with the submuscular implant. Certainly, by recognizing TS early, an earlier resolving intervention is desirable.

## Conclusion

Twiddler syndrome is a not so rare and serious complication of CIED implantation, leading to device malfunctioning and higher risk of infection of the pocket due to multiple re-interventions. In these two cases, different surgical solutions were performed, both resulting to be effective to solve the effects of TS. Psychotherapy seems to be a reasonable preventive approach in patients that have risk factors for developing a TS and that are undergoing an implant of CIED. Furthermore, in the case of previously diagnosed psychiatric disease, an initial psychiatric evaluation is recommended to establish the need to start or optimize oral therapy. In the case of our patients, this preventive approach was not applied due to the absence of obvious psychiatric symptoms before implantation, but it was not even possible after the diagnosis given the poor compliance of patients and family members.

## Supplementary Material

ytae004_Supplementary_Data

## Data Availability

All data are incorporated into the article and its online Supplementary material.

## Lead author biography

Roberta Montisci was born in Cagliari on 5 September 1963. She graduated in October 1988. In 1992, she has a Postgraduate Diploma in Cardiology at the University of Cagliari. Since February 1991, she has been working at the Cardiology-UTIC Department of the University of Cagliari, Monserrato Polyclinic, AOU Cagliari. From December 2019 to date, she has been an Associate Professor of Cardiology at the University of Cagliari. From November 2021 to today, she has been the Director of the Department Cardiology-UTIC of the AOU of Cagliari. The scientific communications in which Professor Roberta Montisci participated as author and co-author have been presented in numerous congresses.

## Supplementary material

**Consent:** The authors confirm that written, informed consent for submission and publication of this case series, including images and associated text, has been obtained from the participants in line with COPE guidance. All participants were 18 years of age at the time of consent.

**Funding:** None declared.
