# Supplementary material for: A case series of the twiddler syndrome
Source: Eur Heart J Case Rep. 2024 Jan 5;8(1):ytae004. doi: 10.1093/ehjcr/ytae004 (PMC10794872; doi:10.1093/ehjcr/ytae004)
Supplement: ytae004_Supplementary_Data [file ytae004_supplementary_data.pptx]

## Slide 1
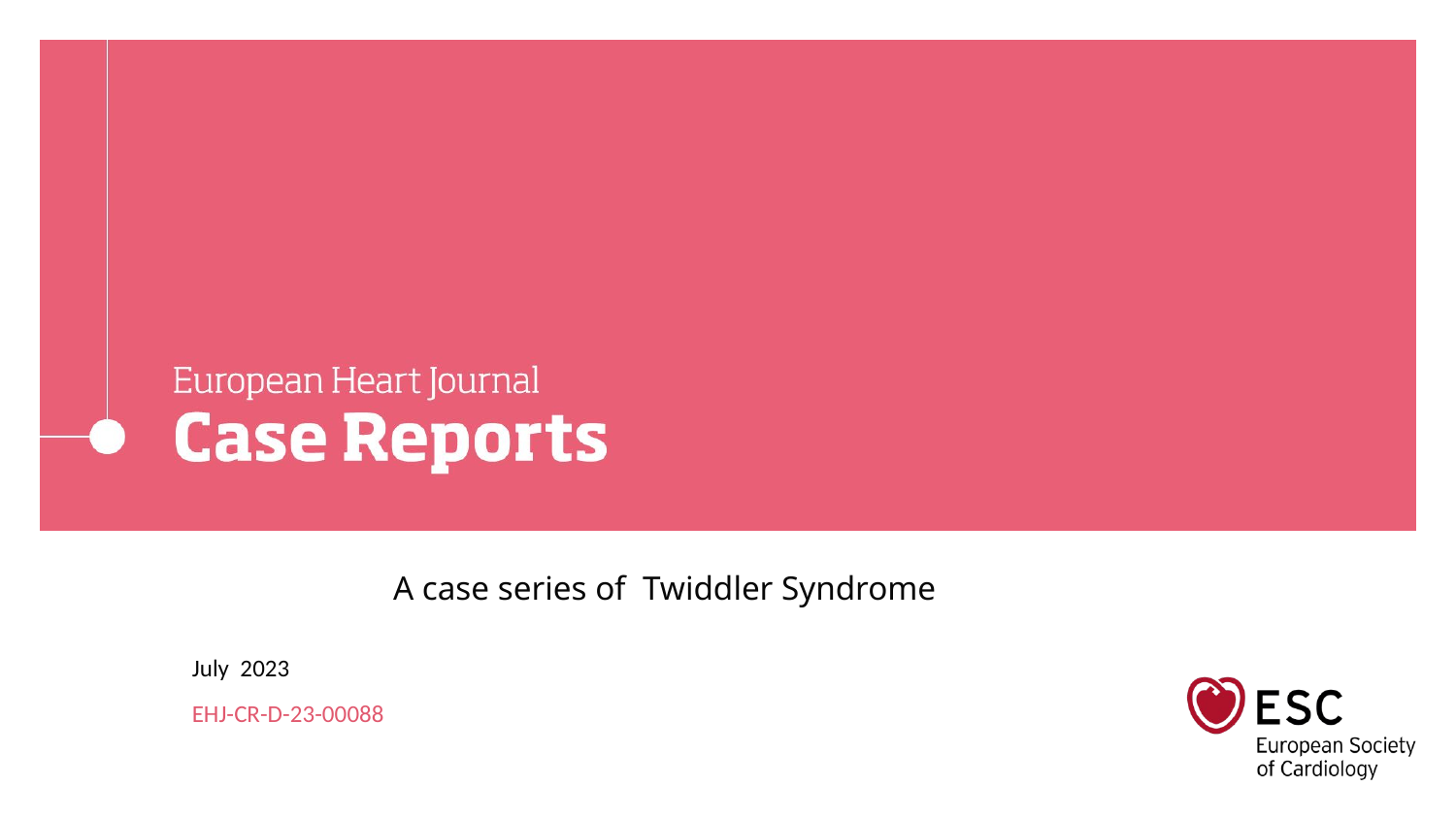

# A case series of Twiddler Syndrome
July 2023
EHJ-CR-D-23-00088

## Slide 2
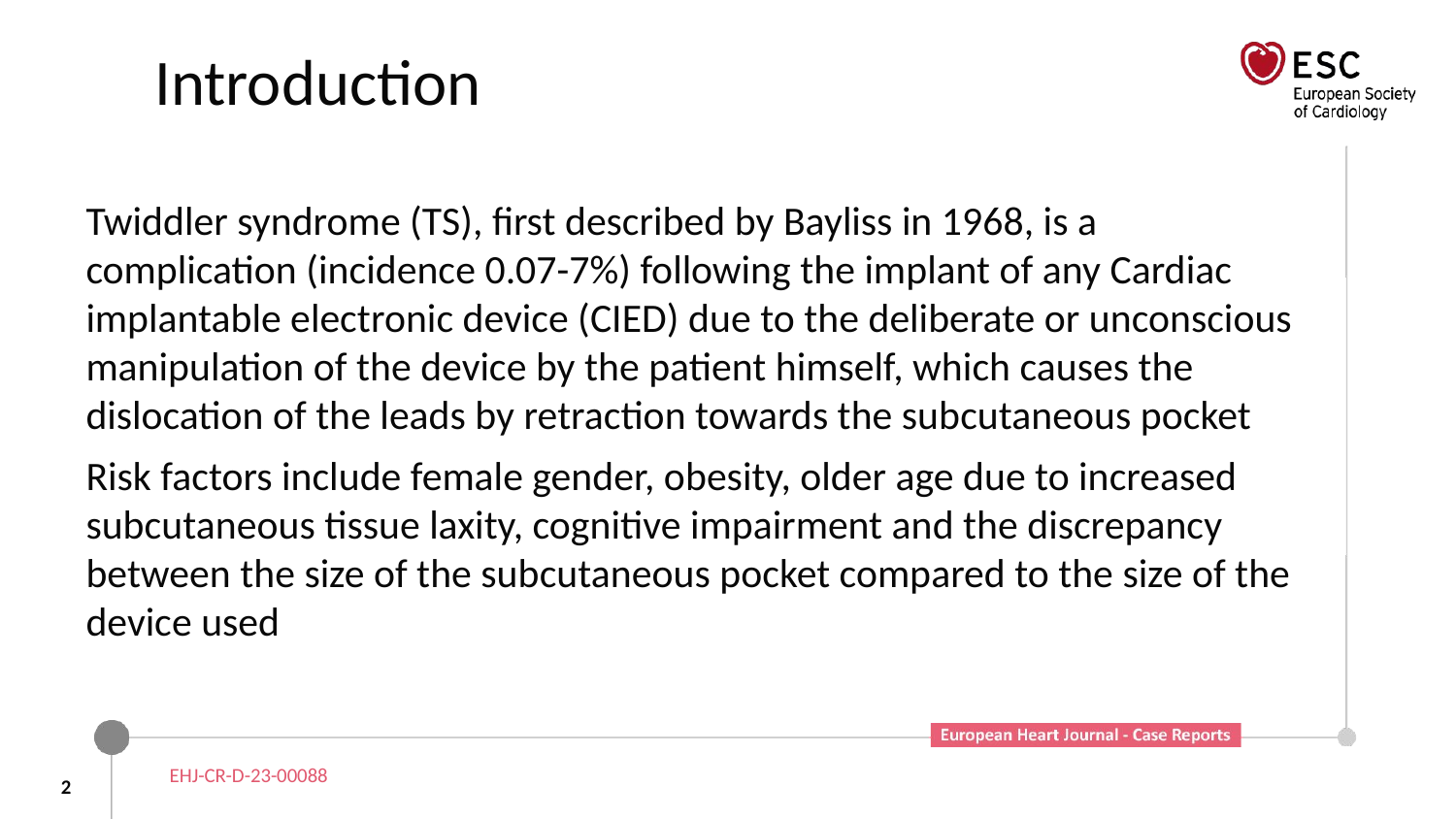

# Introduction
Twiddler syndrome (TS), first described by Bayliss in 1968, is a complication (incidence 0.07-7%) following the implant of any Cardiac implantable electronic device (CIED) due to the deliberate or unconscious manipulation of the device by the patient himself, which causes the dislocation of the leads by retraction towards the subcutaneous pocket
Risk factors include female gender, obesity, older age due to increased subcutaneous tissue laxity, cognitive impairment and the discrepancy between the size of the subcutaneous pocket compared to the size of the device used
2
EHJ-CR-D-23-00088

## Slide 3
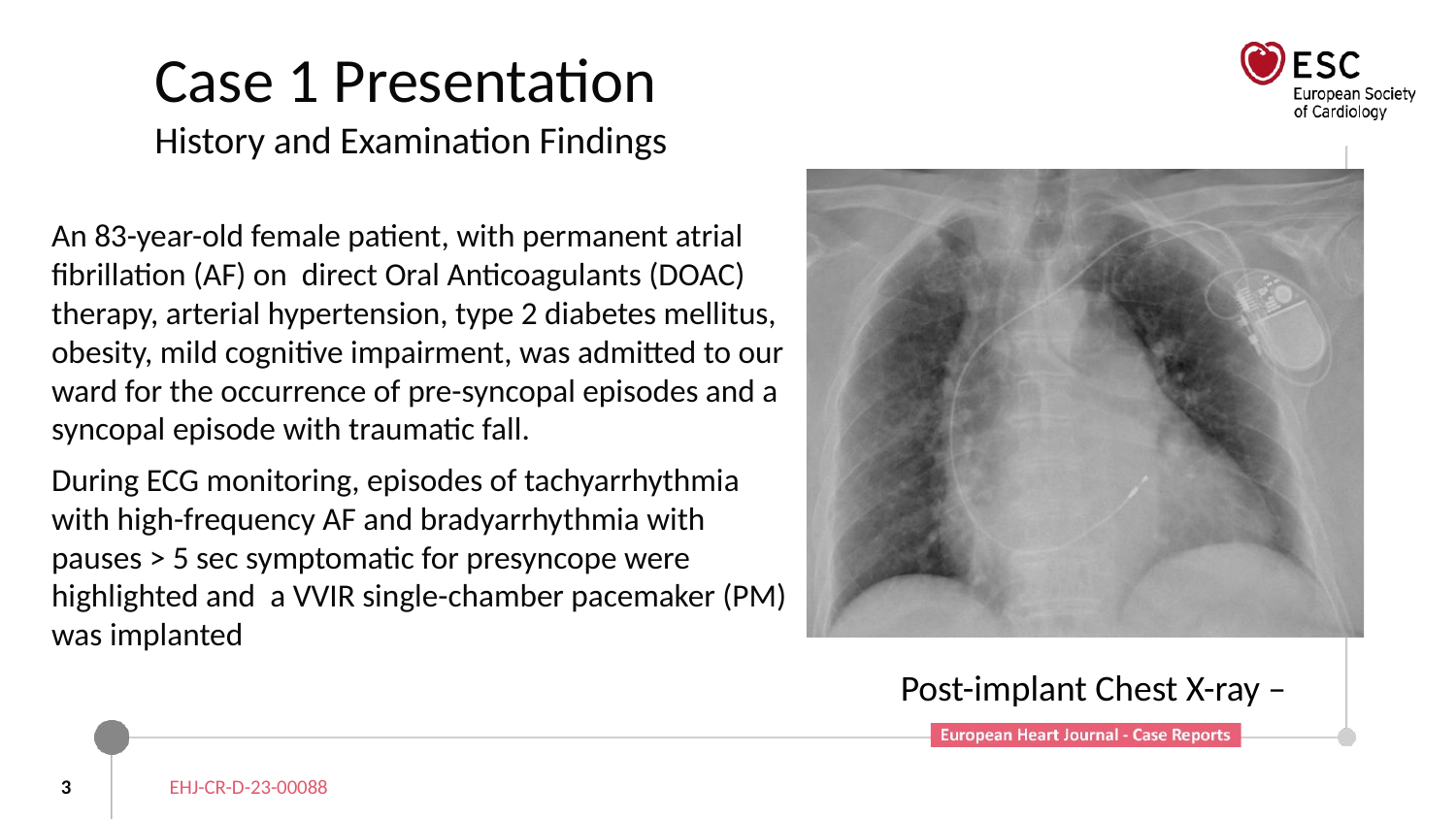

# Case 1 Presentation History and Examination Findings
An 83-year-old female patient, with permanent atrial fibrillation (AF) on direct Oral Anticoagulants (DOAC) therapy, arterial hypertension, type 2 diabetes mellitus, obesity, mild cognitive impairment, was admitted to our ward for the occurrence of pre-syncopal episodes and a syncopal episode with traumatic fall.
During ECG monitoring, episodes of tachyarrhythmia with high-frequency AF and bradyarrhythmia with pauses > 5 sec symptomatic for presyncope were highlighted and a VVIR single-chamber pacemaker (PM) was implanted
Post-implant Chest X-ray –
3
EHJ-CR-D-23-00088

## Slide 4
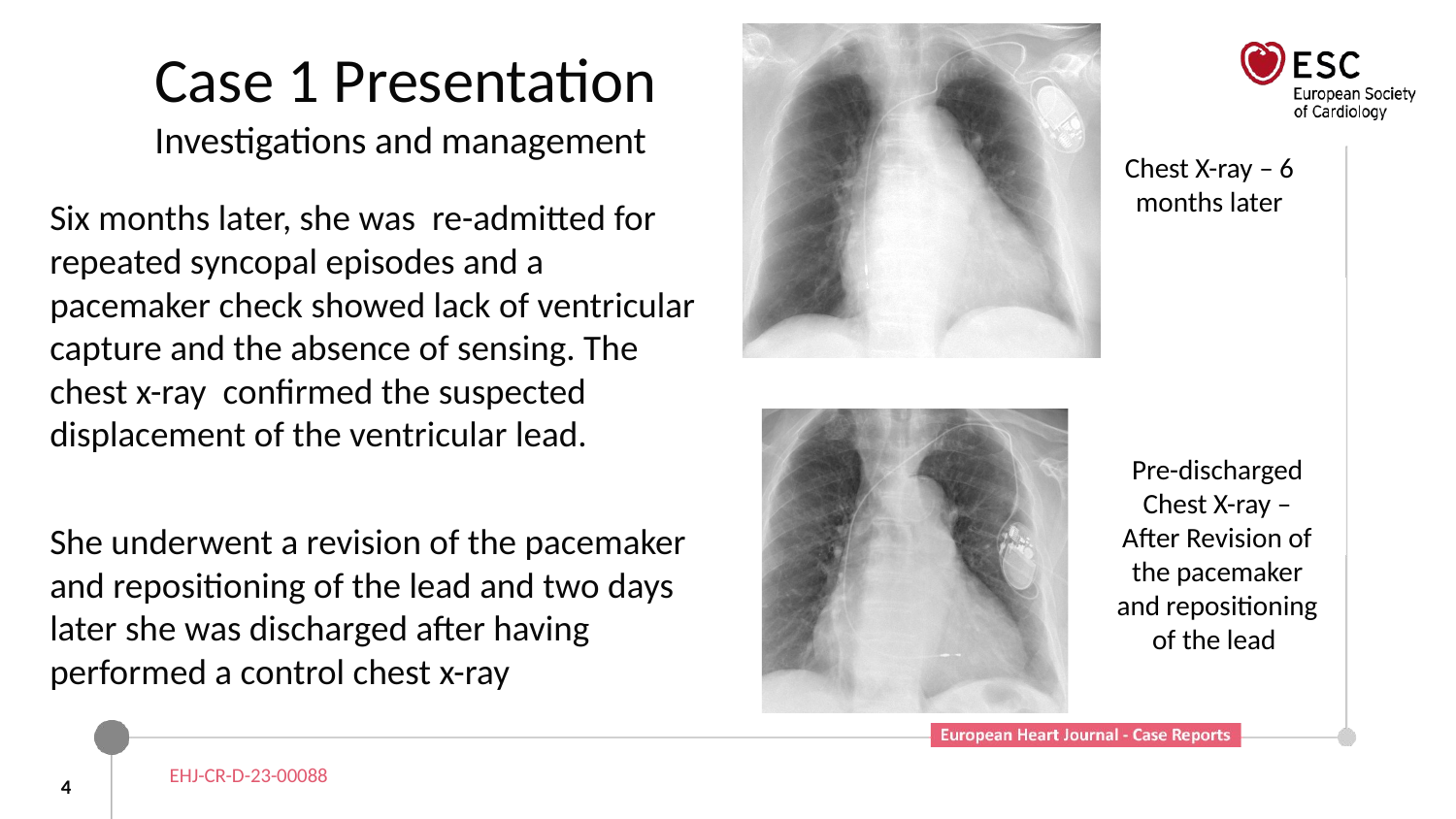

# Case 1 Presentation Investigations and management
Chest X-ray – 6 months later
Six months later, she was re-admitted for repeated syncopal episodes and a pacemaker check showed lack of ventricular capture and the absence of sensing. The chest x-ray confirmed the suspected displacement of the ventricular lead.
She underwent a revision of the pacemaker and repositioning of the lead and two days later she was discharged after having performed a control chest x-ray
Pre-discharged Chest X-ray – After Revision of the pacemaker and repositioning of the lead
4
EHJ-CR-D-23-00088

## Slide 5
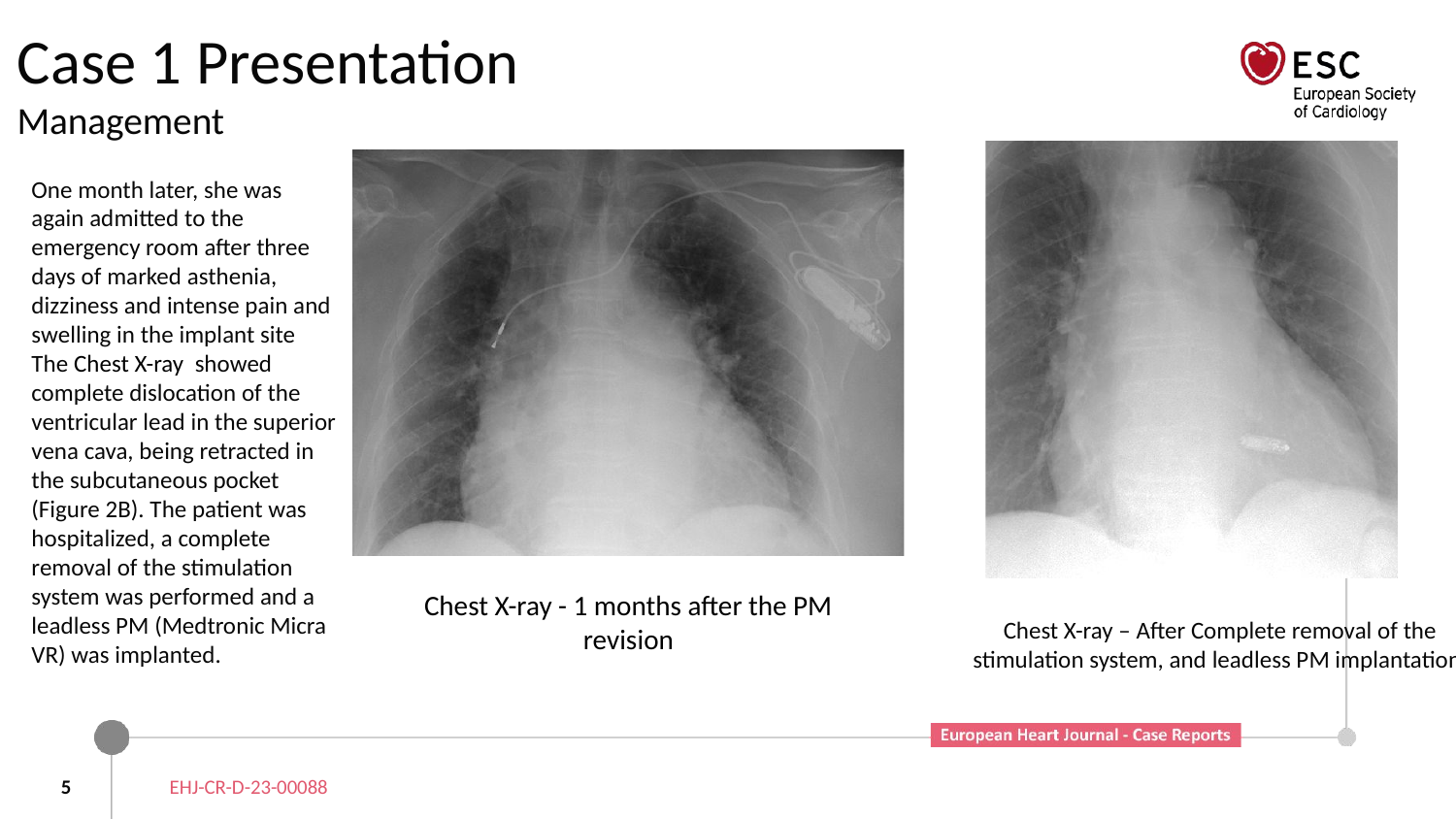

# Case 1 Presentation Management
One month later, she was again admitted to the emergency room after three days of marked asthenia, dizziness and intense pain and swelling in the implant site
The Chest X-ray showed complete dislocation of the ventricular lead in the superior vena cava, being retracted in the subcutaneous pocket (Figure 2B). The patient was hospitalized, a complete removal of the stimulation system was performed and a leadless PM (Medtronic Micra VR) was implanted.
Chest X-ray - 1 months after the PM revision
Chest X-ray – After Complete removal of the stimulation system, and leadless PM implantation
5
EHJ-CR-D-23-00088

## Slide 6
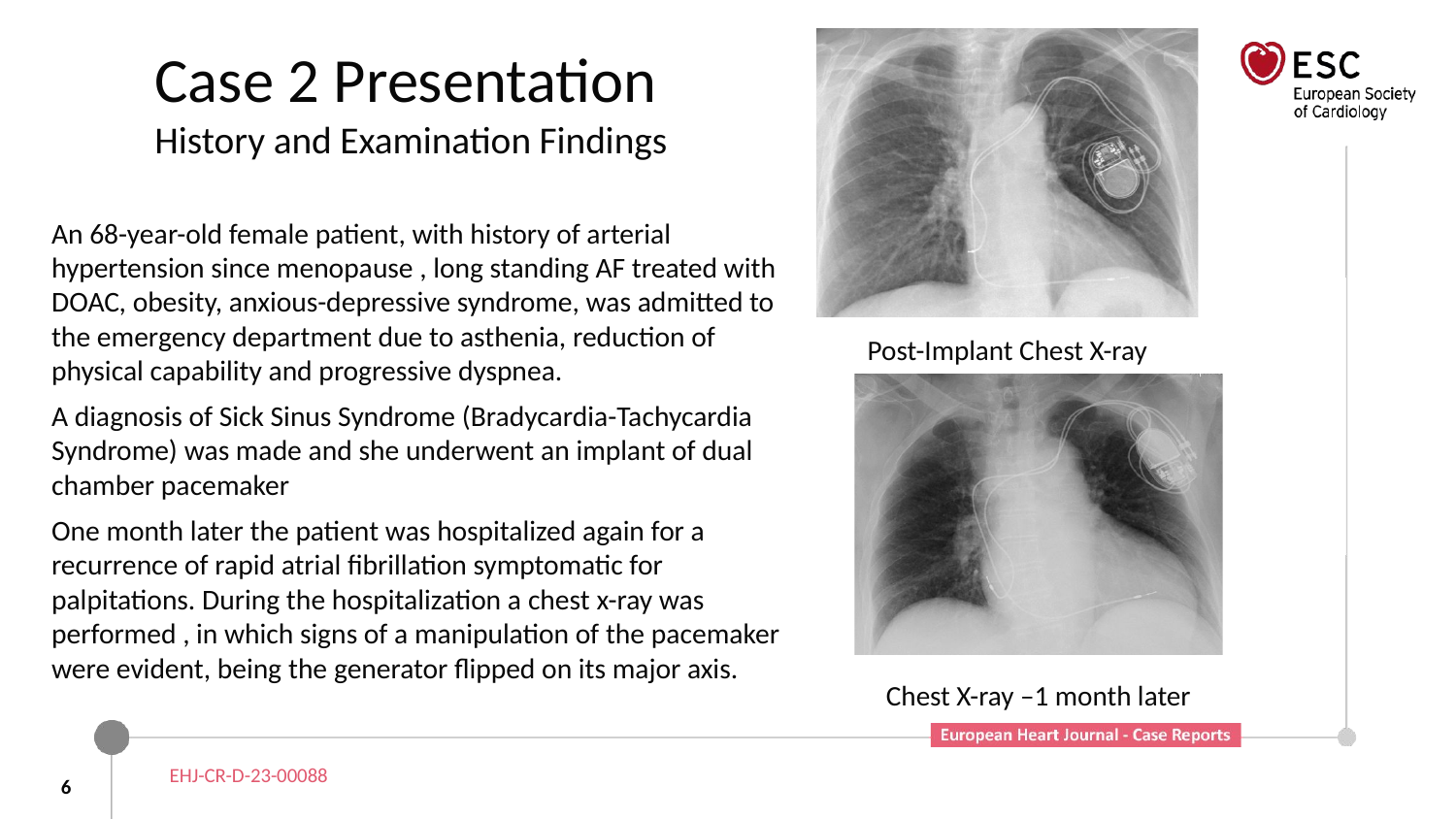

# Case 2 Presentation History and Examination Findings
An 68-year-old female patient, with history of arterial hypertension since menopause , long standing AF treated with DOAC, obesity, anxious-depressive syndrome, was admitted to the emergency department due to asthenia, reduction of physical capability and progressive dyspnea.
A diagnosis of Sick Sinus Syndrome (Bradycardia-Tachycardia Syndrome) was made and she underwent an implant of dual chamber pacemaker
One month later the patient was hospitalized again for a recurrence of rapid atrial fibrillation symptomatic for palpitations. During the hospitalization a chest x-ray was performed , in which signs of a manipulation of the pacemaker were evident, being the generator flipped on its major axis.
Post-Implant Chest X-ray
Chest X-ray –1 month later
6
EHJ-CR-D-23-00088

## Slide 7
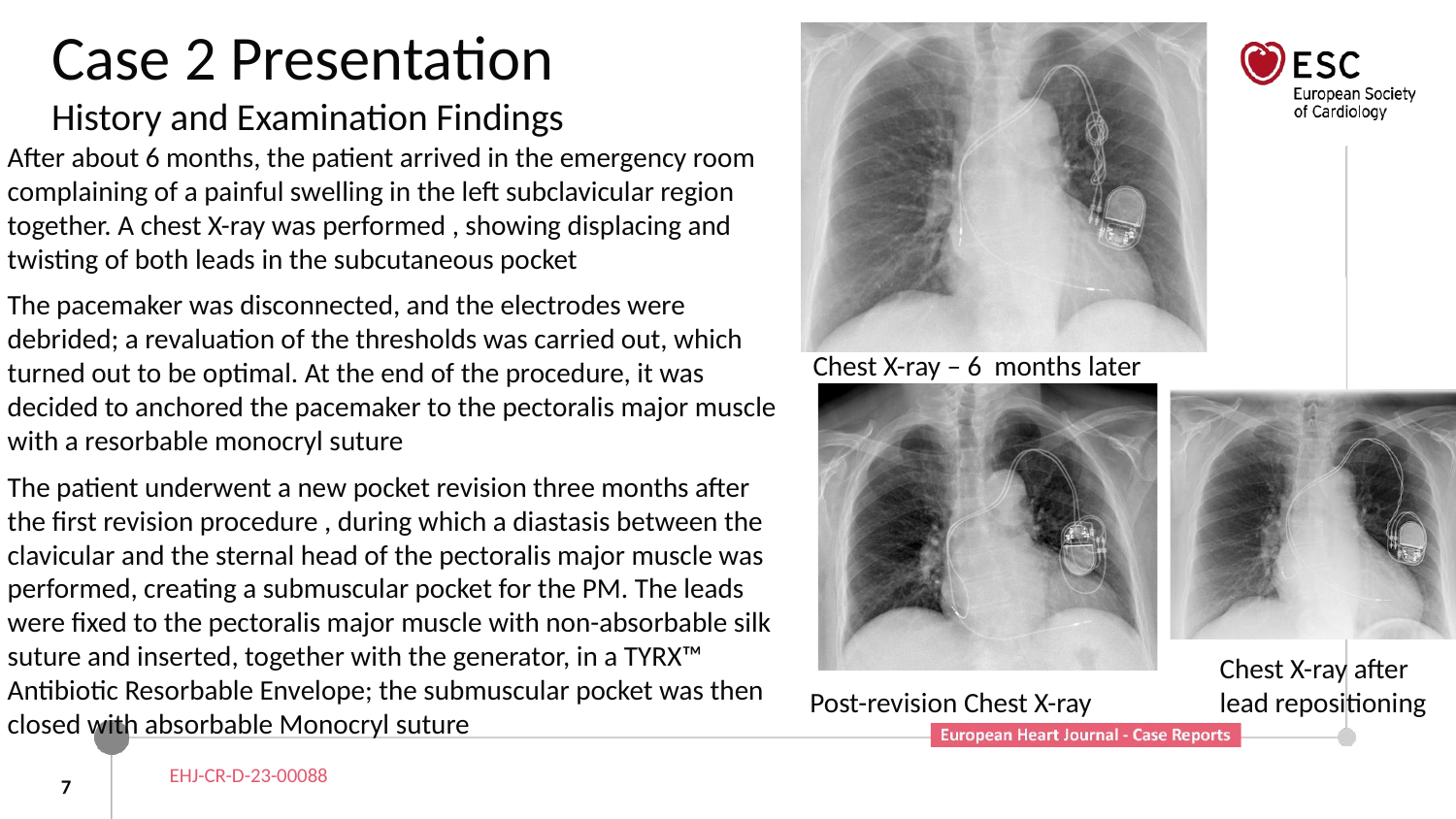

# Case 2 Presentation History and Examination Findings
After about 6 months, the patient arrived in the emergency room complaining of a painful swelling in the left subclavicular region together. A chest X-ray was performed , showing displacing and twisting of both leads in the subcutaneous pocket
The pacemaker was disconnected, and the electrodes were debrided; a revaluation of the thresholds was carried out, which turned out to be optimal. At the end of the procedure, it was decided to anchored the pacemaker to the pectoralis major muscle with a resorbable monocryl suture
The patient underwent a new pocket revision three months after the first revision procedure , during which a diastasis between the clavicular and the sternal head of the pectoralis major muscle was performed, creating a submuscular pocket for the PM. The leads were fixed to the pectoralis major muscle with non-absorbable silk suture and inserted, together with the generator, in a TYRX™ Antibiotic Resorbable Envelope; the submuscular pocket was then closed with absorbable Monocryl suture
Chest X-ray – 6 months later
Chest X-ray after
lead repositioning
Post-revision Chest X-ray
7
EHJ-CR-D-23-00088

## Slide 8
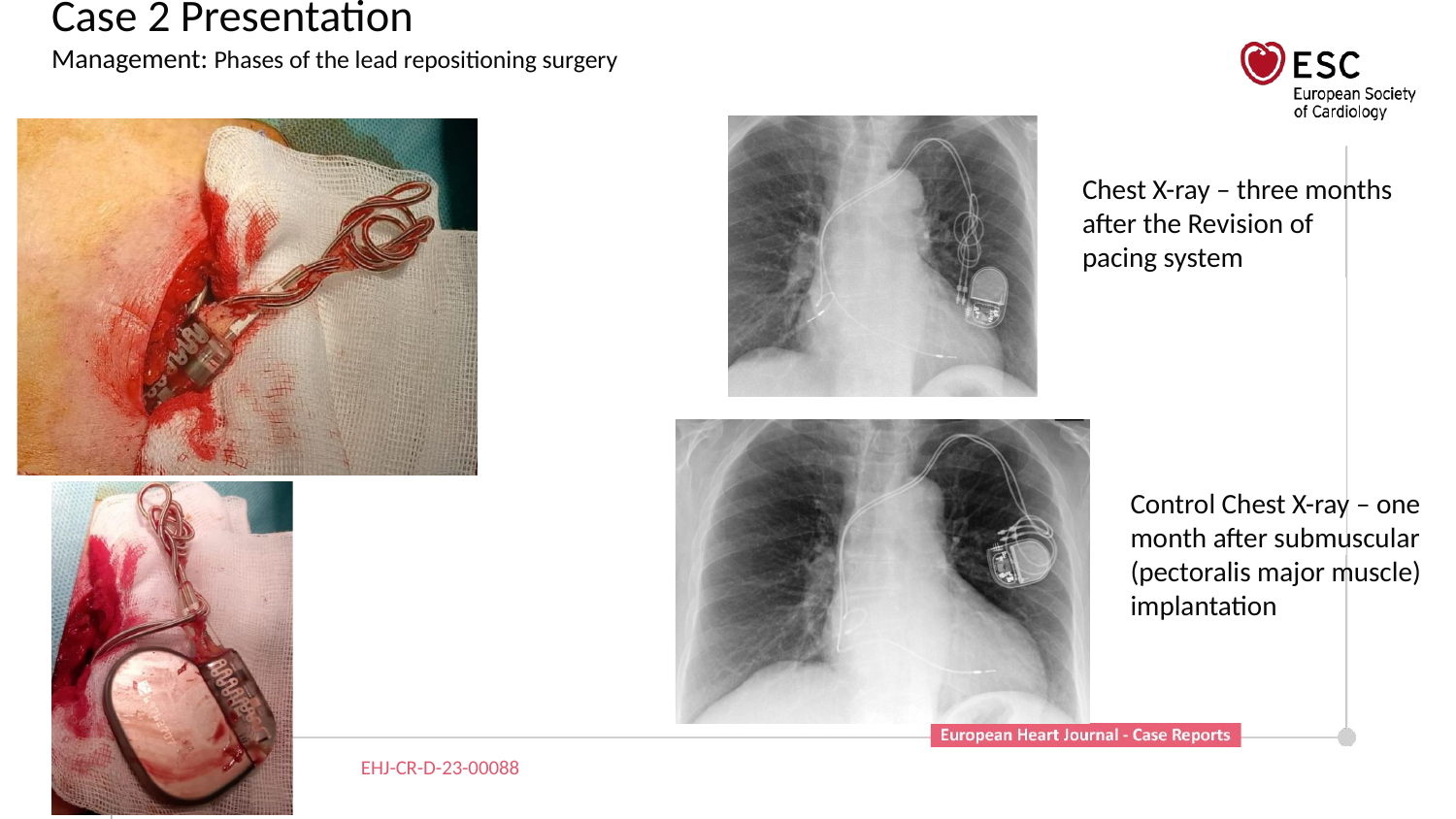

# Case 2 Presentation Management: Phases of the lead repositioning surgery
Chest X-ray – three months after the Revision of pacing system
Control Chest X-ray – one month after submuscular (pectoralis major muscle) implantation
EHJ-CR-D-23-00088
8

## Slide 9
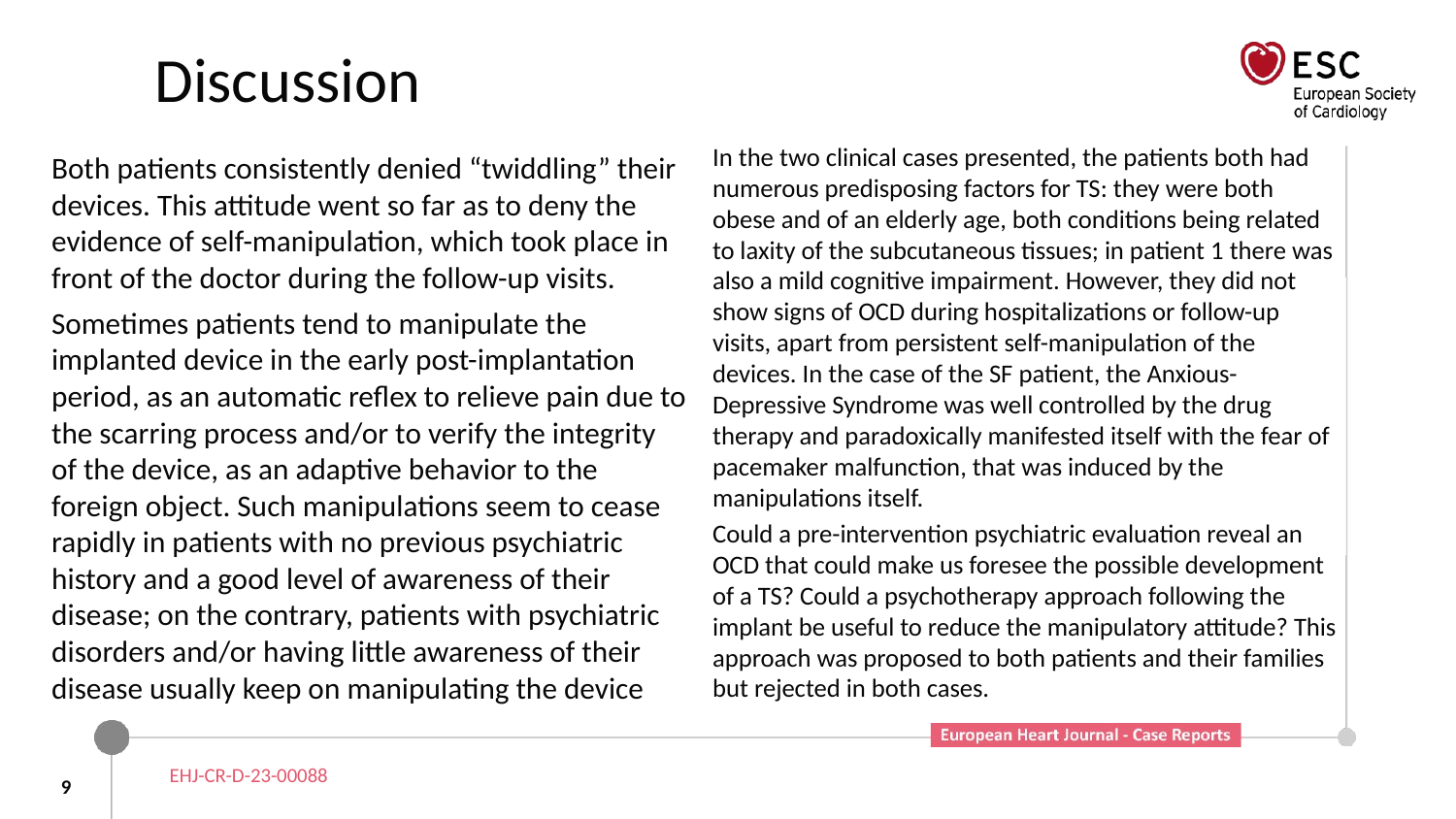

# Discussion
In the two clinical cases presented, the patients both had numerous predisposing factors for TS: they were both obese and of an elderly age, both conditions being related to laxity of the subcutaneous tissues; in patient 1 there was also a mild cognitive impairment. However, they did not show signs of OCD during hospitalizations or follow-up visits, apart from persistent self-manipulation of the devices. In the case of the SF patient, the Anxious-Depressive Syndrome was well controlled by the drug therapy and paradoxically manifested itself with the fear of pacemaker malfunction, that was induced by the manipulations itself.
Could a pre-intervention psychiatric evaluation reveal an OCD that could make us foresee the possible development of a TS? Could a psychotherapy approach following the implant be useful to reduce the manipulatory attitude? This approach was proposed to both patients and their families but rejected in both cases.
Both patients consistently denied “twiddling” their devices. This attitude went so far as to deny the evidence of self-manipulation, which took place in front of the doctor during the follow-up visits.
Sometimes patients tend to manipulate the implanted device in the early post-implantation period, as an automatic reflex to relieve pain due to the scarring process and/or to verify the integrity of the device, as an adaptive behavior to the foreign object. Such manipulations seem to cease rapidly in patients with no previous psychiatric history and a good level of awareness of their disease; on the contrary, patients with psychiatric disorders and/or having little awareness of their disease usually keep on manipulating the device
9
EHJ-CR-D-23-00088

## Slide 10
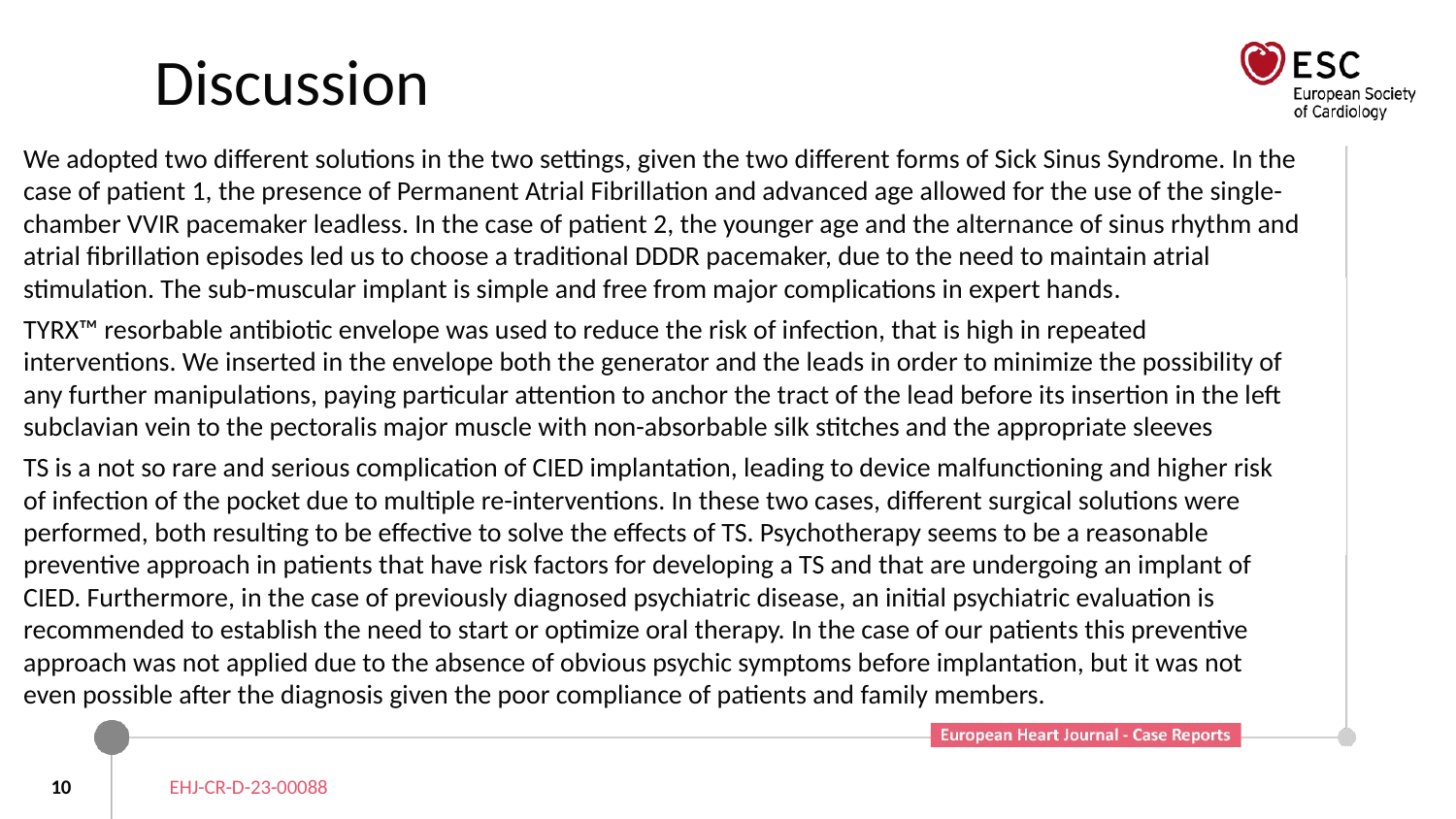

# Discussion
We adopted two different solutions in the two settings, given the two different forms of Sick Sinus Syndrome. In the case of patient 1, the presence of Permanent Atrial Fibrillation and advanced age allowed for the use of the single-chamber VVIR pacemaker leadless. In the case of patient 2, the younger age and the alternance of sinus rhythm and atrial fibrillation episodes led us to choose a traditional DDDR pacemaker, due to the need to maintain atrial stimulation. The sub-muscular implant is simple and free from major complications in expert hands.
TYRX™ resorbable antibiotic envelope was used to reduce the risk of infection, that is high in repeated interventions. We inserted in the envelope both the generator and the leads in order to minimize the possibility of any further manipulations, paying particular attention to anchor the tract of the lead before its insertion in the left subclavian vein to the pectoralis major muscle with non-absorbable silk stitches and the appropriate sleeves
TS is a not so rare and serious complication of CIED implantation, leading to device malfunctioning and higher risk of infection of the pocket due to multiple re-interventions. In these two cases, different surgical solutions were performed, both resulting to be effective to solve the effects of TS. Psychotherapy seems to be a reasonable preventive approach in patients that have risk factors for developing a TS and that are undergoing an implant of CIED. Furthermore, in the case of previously diagnosed psychiatric disease, an initial psychiatric evaluation is recommended to establish the need to start or optimize oral therapy. In the case of our patients this preventive approach was not applied due to the absence of obvious psychic symptoms before implantation, but it was not even possible after the diagnosis given the poor compliance of patients and family members.
10
EHJ-CR-D-23-00088

## Slide 11
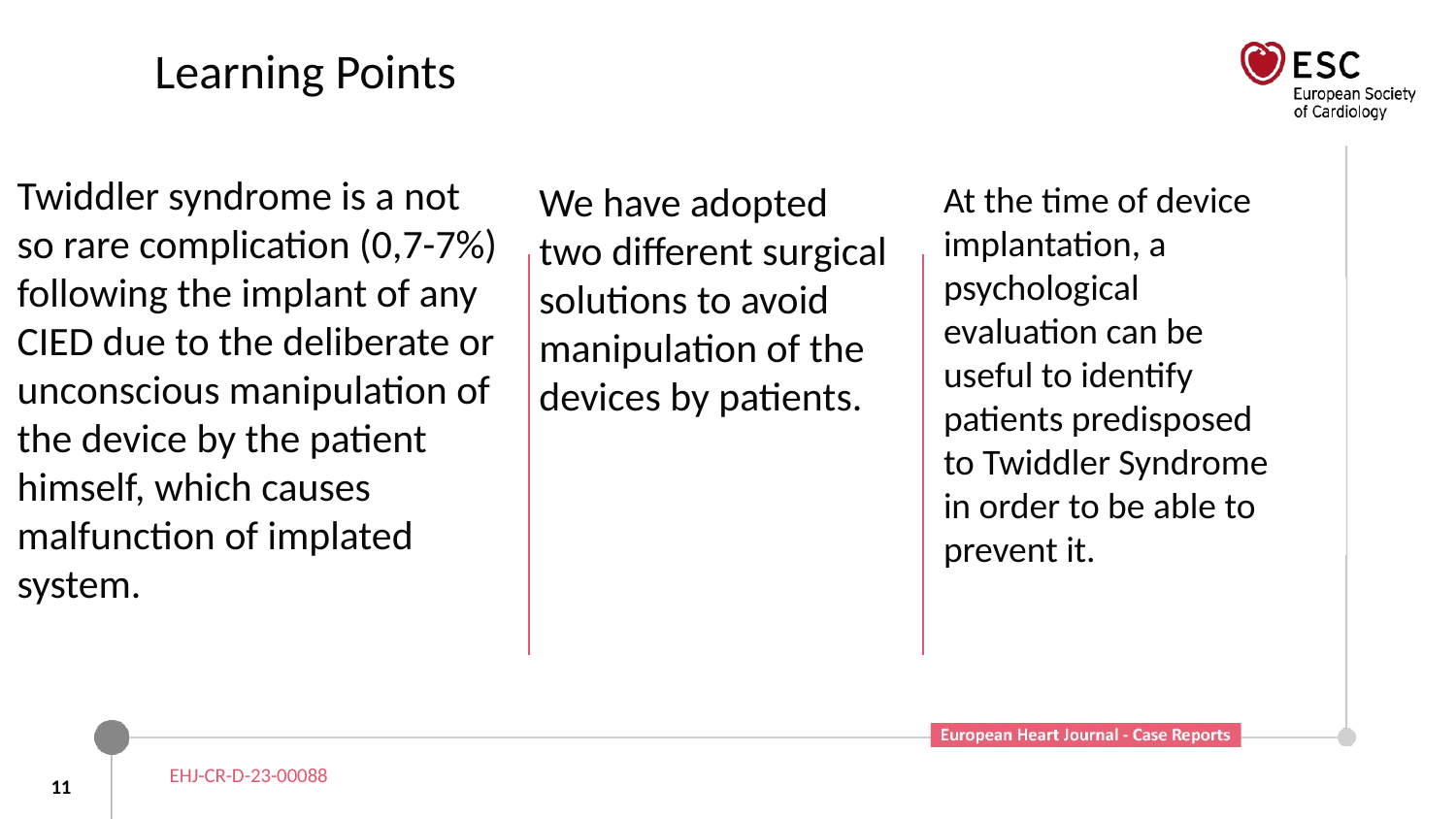

# Learning Points
Twiddler syndrome is a not so rare complication (0,7-7%) following the implant of any CIED due to the deliberate or unconscious manipulation of the device by the patient himself, which causes malfunction of implated system.
We have adopted two different surgical solutions to avoid manipulation of the
devices by patients.
At the time of device implantation, a psychological evaluation can be useful to identify patients predisposed to Twiddler Syndrome in order to be able to prevent it.
11
EHJ-CR-D-23-00088

## Slide 12
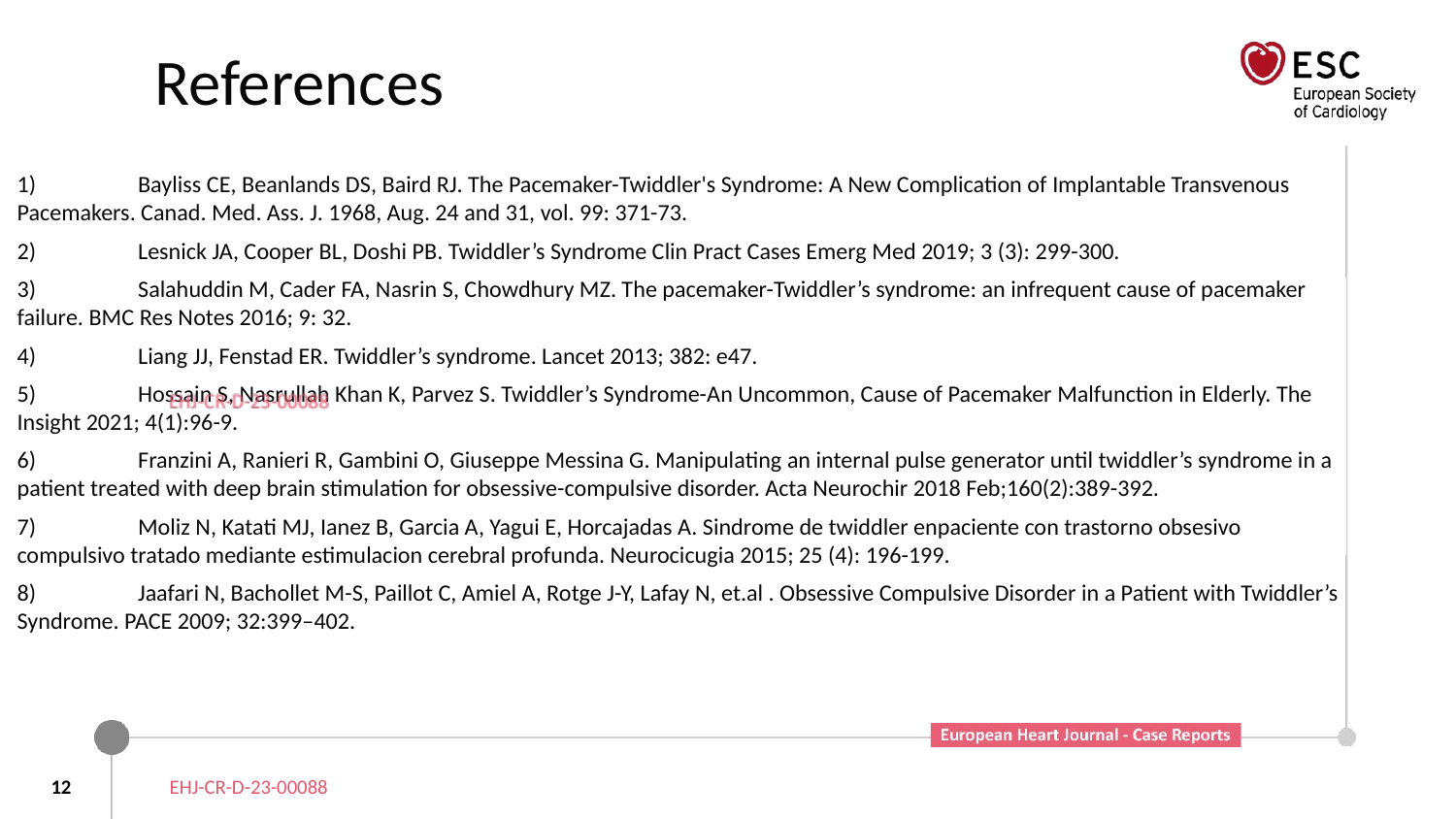

# References
1)	Bayliss CE, Beanlands DS, Baird RJ. The Pacemaker-Twiddler's Syndrome: A New Complication of Implantable Transvenous Pacemakers. Canad. Med. Ass. J. 1968, Aug. 24 and 31, vol. 99: 371-73.
2)	Lesnick JA, Cooper BL, Doshi PB. Twiddler’s Syndrome Clin Pract Cases Emerg Med 2019; 3 (3): 299-300.
3)	Salahuddin M, Cader FA, Nasrin S, Chowdhury MZ. The pacemaker-Twiddler’s syndrome: an infrequent cause of pacemaker failure. BMC Res Notes 2016; 9: 32.
4)	Liang JJ, Fenstad ER. Twiddler’s syndrome. Lancet 2013; 382: e47.
5)	Hossain S, Nasrullah Khan K, Parvez S. Twiddler’s Syndrome-An Uncommon, Cause of Pacemaker Malfunction in Elderly. The Insight 2021; 4(1):96-9.
6)	Franzini A, Ranieri R, Gambini O, Giuseppe Messina G. Manipulating an internal pulse generator until twiddler’s syndrome in a patient treated with deep brain stimulation for obsessive-compulsive disorder. Acta Neurochir 2018 Feb;160(2):389-392.
7)	Moliz N, Katati MJ, Ianez B, Garcia A, Yagui E, Horcajadas A. Sindrome de twiddler enpaciente con trastorno obsesivo compulsivo tratado mediante estimulacion cerebral profunda. Neurocicugia 2015; 25 (4): 196-199.
8)	Jaafari N, Bachollet M-S, Paillot C, Amiel A, Rotge J-Y, Lafay N, et.al . Obsessive Compulsive Disorder in a Patient with Twiddler’s Syndrome. PACE 2009; 32:399–402.
12
EHJ-CR-D-23-00088
